# A comparative retrospective analysis on robot-assisted laparoscopic surgery compared to conventional laparoscopy in case of myomectomy: experience in a third-level hospital of Southern Italy

**DOI:** 10.1007/s13304-024-01863-x

**Published:** 2024-04-30

**Authors:** Luigi Della Corte, Giuseppe D’Angelo, Mario Ascione, Marcello Granata, Pierluigi Giampaolino, Attilio Di Spiezio Sardo, Giuseppe Bifulco

**Affiliations:** 1 Department of Neuroscience, Reproductive Sciences and Dentistry, School of Medicine, University of Naples Federico II, Via Sergio Pansini 5, 80131 Naples, Italy; 2 Department of Public Health, University of Naples Federico II, Naples, Italy

**Keywords:** Uterine fibroids, Robotic-assisted laparoscopic surgery, Laparoscopy, Myomectomy

## Abstract

Uterine myomas are the most common gynecological disease in reproductive-aged women, present several symptoms, and require effective medical and/or surgical strategies. This study aimed to compare robotic-assisted laparoscopic myomectomy (RALM) with laparoscopic myomectomy (LM) in terms of operative times, intraoperative estimated blood loss, pre- and post-hemoglobin levels drop, and length of hospital stay. Data from 50 clinical records (25 RALM in Group A and 25 LM in Group B) of patients with uterine fibroids were collected from December 2022 to December 2023 at Gynecological Unit of DAI Materno-Infantile Federico II in Naples, Italy. Patients aged 30–49 years with symptomatic fibroids were included. Data on peri-operative outcomes, including operative time for myomectomy (OTM), overall operative time (OOT), intraoperative estimated blood loss (EBL), pre- and post-operative hemoglobin levels, and length of hospital stay were analyzed. The OTM in the presence of > 5 myomas was 59 [52–65] vs 69 min [61–96] (*p* < 0.001) for RALM and LM groups, respectively. Moreover, also in presence of ≤ 5 myomas, a difference was observed in the RALM group 48[43–55] compared to the LM group 53[50–61] min (*p* = 0.07). The OOT was also statistically significant for Group A compared to Group B (83[65–93] vs 72[56–110] min, *p* < 0.001). There were no significant differences between the two groups in terms of pre- and post-operative hemoglobin levels and EBL (*p* = 0.178). Group A demonstrated a notably shorter hospital stay 1.2 [1–2] days compared to Group B 2.9[3–3.75] days (*p* = 0.007). Our study suggests potential advantages of RALM over LM in terms of reduced operative times and shorter hospital stays. The standardized approach and extensive surgical experience likely contributed to the favorable outcomes of RALM.

## Introduction

Uterine fibroids, also referred to as uterine leiomyomas, are non-cancerous growths of smooth muscle in the uterus that commonly affect women during their reproductive age [1, 2]. The most prevalent symptom is heavy menstrual bleeding, often causing anemia, fatigue, and painful menstrual periods, but patients can complain non-cyclic abdominal pain, discomfort during intercourse, pelvic pressure, urinary incontinence or retention, as well as pain or constipation [3, 4]. Reproductive challenges, including impaired fertility [5], complications during pregnancy, pregnancy loss, and adverse outcomes during childbirth, have also been associated with uterine fibroids [6–8]. Despite its high prevalence, the etiology, pathogenesis, and optimal management, uterine fibromyomatosis remain subjects of ongoing research and debate. Uterine fibroids represent the most common reason for hysterectomy in women. However, the actual frequency may be underestimated, since many cases are either asymptomatic or display insidious symptom development, leading to undiagnosed instances [9].

The identification of uterine fibroids is typically accomplished with ultrasound examination, magnetic resonance imaging (MRI), and/or hysteroscopy, especially for myomas with an important submucosal component [3].

Medical interventions for uterine fibromatosis commonly include estroprogestins, progestins, or GnRH analogs [10, 11]. Considering surgical options, the choice is influenced by several factors such as the patient's age, fertility preservation goals, and patient’s choice. Treatment approaches vary based on the fibroid's location, with hysteroscopic myomectomy [12, 13], laparotomic, or laparoscopic/robotic myomectomy.

In cases of submucosal myomas, the importance of hysteroscopy as a diagnostic tool before planning myomectomy cannot be underestimated. This procedure enables precise visualization and assessment of the extent and location of the myomas within the uterine cavity, guiding surgical decision-making and optimizing patient outcomes especially in those with a previous history of infertility [14].

Presently, open myomectomy is recommended for cases involving large uterine fibroids (> 10 cm) leading to heavy menstrual bleeding and pelvic pain, as well as instances of multiple fibroids [15]. The laparoscopic approach is gaining popularity due to its advantages in minimizing post-operative complications and reducing hospital stays [16, 17].

The robotic approach, perceived as conferring notable advantages to myomectomy, notably in terms of enhanced precision, has long been associated with higher costs. However, with the continuous evolution of technologies and the increasingly widespread adoption of robotic platforms, the economic dynamics of this approach are undergoing a transformative shift. Presently, the robotic myomectomy procedure is witnessing a trend toward reduced costs, marking a departure from its historical characterization as a more expensive alternative [18].

The primary objective of our study was to compare the robotic surgical approach with the laparoscopic one in terms of operative time (for myomectomy and overall), and the secondary outcomes included post-operative hemoglobin levels, estimated blood loss, and length of hospital stay.

## Materials and methods

From December 2022 to December 2023, we retrospectively collected data on 54 patients who underwent robotic-assisted laparoscopic myomectomy (RALM) with the da Vinci Xi surgical system (Intuitive Surgical, USA) (26) compared to those who underwent laparoscopic myomectomy (LM) (28) in the Gynecological Unit of DAI Materno-Infantile of Azienda Ospedaliera Universitaria Federico II in Naples, Italy. All patients aged between 34 and 49 years old and a clinical and ultrasonographic and/or MRI diagnosis of intramural/subserous uterine fibroids (FIGO 3–6 Leiomyoma Subclassification System) [19] (single or multiple fibroids) with symptoms such as menorrhagia and/or pelvic pain. Exclusion criteria were: age > 50 years old, oncological disease, high anesthetic risk (ASA 3–4), fibroid size larger than 10 cm that did not allow removal through mini-invasive surgery (MIS), significantly enlarged uterus reaching the transverse umbilical line and ongoing pregnancy. The patients’ medical records were retrieved and those who met the inclusion criteria were included in the study.

The diagnosis of uterine fibroids was made using a GE Voluson E8 Ultrasound Machine. In cases of diagnostic uncertainty, particularly when signs suggestive of bladder or bowel compression or significant vascularity were present, additional diagnostic investigation were pursued through abdominal and pelvic MRI with and without contrast to provide further clarity and insight into the underlying disease. All patients were appropriately counseled and written informed consent was obtained. All cases were performed by two surgeons skilled in laparoscopic as well as robotic surgery. All patients received a mechanical bowel preparation, peri-operative antibiotics, and compression stockings where necessary. A Foley catheter was inserted in the bladder and a uterine manipulator was placed.

**Fig. 1:**
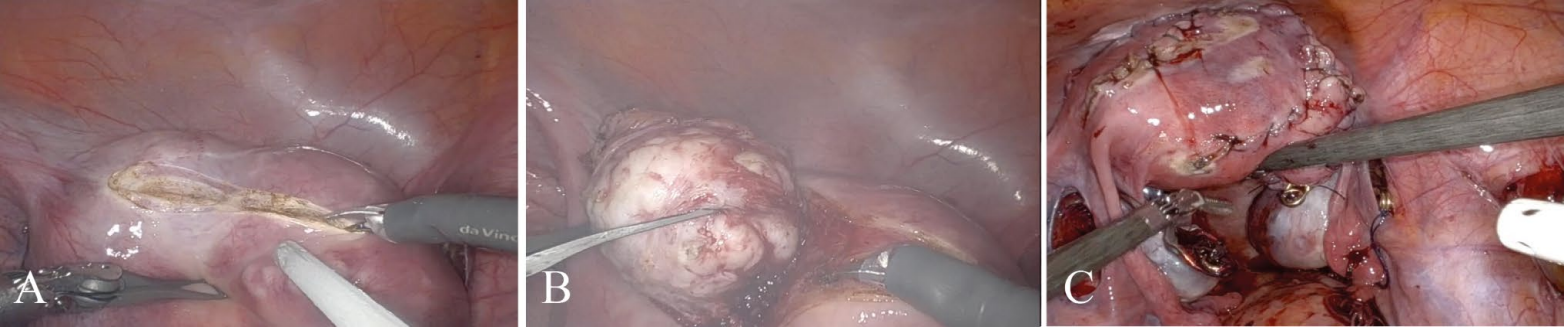
**A**–**C** The surgical steps for robotic-assisted laparoscopic myomectomy were illustrated. **A** Incision on the uterine serosa overlying the myoma; **B** enucleation of the myoma; **C** closure of the uterine surgical defect in multiple layers

**Fig. 2:**
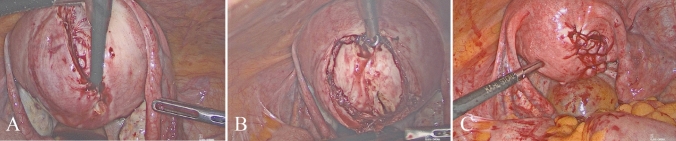
**A**–**C** The surgical steps for laparoscopic myomectomy were illustrated. **A** Incision on the uterine serosa overlying the myoma; **B** enucleation of the myoma; **C** closure of the uterine surgical defect in multiple layers

For RALM procedures after pneumoperitoneum was established, peritoneal access was obtained using an 8-mm trocar placed through the umbilicus or above the umbilicus according to the size of the fibroid. The left robotic instrument port was inserted 10 cm lateral to the camera port at the mid-clavicular line and 2–3 cm below the camera port. The right robotic instrument port point was symmetrically on the contralateral side of the left robotic port. The assistant port was introduced between the left robotic port and the camera port, approximately 2–3 cm above the camera port with the AirSeal^®^ system connected (Conmed, USA). The sizes of trocars for EndoWrist equipment and assistant port were 8 mm and 12 mm, respectively. The third robotic instrument port was not used. After the docking procedure, the camera and system's EndoWrist instruments were introduced through trocars. The surgical steps for RALM (Fig. 1) and LM were similar (Fig. 2).

The main distinction between the RALM and LM procedures lay in the placement of trocars. In LM, a 10-mm trocar was positioned either through the umbilicus or above it, contingent upon the size of the fibroid. Two 5-mm accessory trocars were inserted in the right and left lower quadrants. An additional 5-mm trocar was placed suprapubically at the midline, positioned below 5–10 cm from the camera port. After the excision of the myomas, the surgical defect in the uterus was meticulously closed using 0 monofilament polyglyconate copolymer (V-LOC Barbed Sutures; Medtronic) in two or three layers. The extraction of the fibroids was performed utilizing both the ExCITE (Extracorporeal Instrument for Tissue Extraction) [20] technique or through a minilaparotomy. The selected approach depended on some factors such as the size of the myomas, their number, and the surgical preference of the operating surgeon.

Perioperative outcomes were analyzed: pre- and post-operative hemoglobin levels, operative time for myomectomy (OTM), overall operative time (OOT), estimated blood loss (EBL), length of hospital stay, and conversion rate to laparotomy.

The OTM included the time enroll to perform the myomectomy from the incision of the uterine serosa to the culmination of the procedure, occurring after the closure of uterine breaches. The EBL was calculated by determining the difference between the volume of the fluid used for irrigation and suction at the end of the procedure, allowing for an estimation of the amount of blood loss during the surgical procedure.

The OOT included a comprehensive duration, spanning from skin preparation, dressing, trocar incisions, CO_2_ insufflation, port placement, exploration, and any supplementary procedures deemed necessary, such as laparoscopic adhesiolysis, myomectomy procedure itself, uterine suture, and subsequent skin closure. None of the myomectomy procedures, whether robotic or laparoscopic, was converted to laparotomy. The peri-operative patient management used reflected ERAS protocols [21].

### Statistical analysis

Concerning the continuous variables, once the normality assumption was assessed using the Shapiro–Wilk test, the medians, first and third quartiles were reported. To test the hypothesis of equality between the two groups, the Wilcoxon test was performed. For categorical variables, the frequencies and their rates were reported. The ANOVA (Analysis of Variance) test was used as a statistical test. Statistical significance was considered with *p* < 0.05.

## Results

A total of 50 patients met the inclusion criteria and were considered in our analysis: 25 patients underwent RALM (Group A), while 25 patients underwent LM procedure (Group B). Four patients were excluded, because the histological examination revealed three cases of adenomyomas and one of sarcoma.

Clinical and demographic patients’ characteristics are summarized in Table 1. The average age was 40 (34–43) years in Group A and 46 (43–49) years old in Group B. A significant difference in BMI was observed between the two groups, with Group A exhibiting a higher BMI 35.1 (31.0–36.3) compared to Group B 31.0 (27.8–32.0). Age and BMI were adjusted for as confounding factors in the examination of variables. All patients included in the analysis had a diagnosis of uterine fibroids at imaging. In Group A, the maximum size of the fibroid was 7 (6–8) cm, while in Group B 5 (4–8) cm, revealing a significant difference between the two groups (*p* < 0.002).

**Table 1:** Demographic and clinical characteristics of each group. Values are expressed as median, first and third quartile

	Group A (*n* = 25)—robotic myomectomy	Group B (*n* = 25)—laparoscopic myomectomy	*P* value
Age	40[34–43]	46[43–49]	< 0.001
BMI	35.1[31.0–36.3]	31.0[27.8–32.0]	0.011
Myoma size (cm)	7[4–9]	5[4–8]	0.002
Surgical Indication and Technique			< 0.001
No of myomas 2–5	10/25(40%)	16/25(64%)	
No of myomas > 5	15/25(60%)	9/25(36%)	

In Group A, a statistically significant difference was observed in the number of myomas compared to Group B (*p* < 0.001), indicating a higher number of myomas in the RALM group. Table 2 illustrates the main and secondary) endpoints. The operative time for myomectomy (OTM) in the presence of more than 5 myomas was 59 (52–65) vs 69 min (61–96) (*p* < 0.001) for RALM and LM groups, respectively. Moreover, also in presence of ≤ 5 myomas, a difference was observed in the RALM group 48 (43–55) compared to the LM group 53(50–61) min (*p* = 0.07). The OOT was also statistically significant for Group A compared to Group B 83 (65–93) vs 72 (56–110) min, (*p* < 0.001). The average estimated blood loss (EBL) for patients undergoing RALM was 100 (90–150) ml, while for those undergoing LM was 115 (100–164) ml: however, this difference was not statistically significant (*p* = 0.178). No significant differences between the two groups in terms of pre- and post-operative hemoglobin levels were noted. Group A demonstrated a notably shorter hospital stay 1.2 (1–2) days compared to Group B 2.9 (3–3.75) days, with a statistical significance difference (*p* = 0.007). The opening of the endometrial cavity during the myomectomy did not occur. No conversion to laparotomy was needed for the two groups.

**Table 2:** Main and secondary outcomes

	Group A (*n* = 25)—Robotic Myomectomy	Group B (*n* = 25)—laparoscopic myomectomy	*P* value
Operative time for myomectomy [OTM] (min)			
Myomas 2–5	48[43–55]	53[50–61]	0.07
Myomas > 5	59[52–65]	69[61–96]	< 0.001
Overall operative time [OOT](min)	83[65–93]	72[56–110]	< 0.001
Pre-operative hemoglobin (g/dl)	12 ± 1.3	11.7 ± 0.9	
Post-operative hemoglobin (g/dl)	11.4 ± 1.3	10.9 ± 1.2	
Estimated blood loss (ml)	100[90–150]	115[100–164]	0.178
Laparatomic conversion	0	0	
Hospital stay (days)	1.2 [1, 2]	2.9 [3–3.75]	< 0.001

Continuous variables are expressed as median, first quartile, and third quartile; categorical variables are expressed as frequency and percentage

## Discussion

Uterine fibroids are benign tumors originating from the proliferation of smooth muscle cells within the uterine wall and represent the most prevalent non-cancerous tumors affecting women [22]. While not all uterine fibroids show symptoms, approximately 30% of affected women has abnormal uterine bleeding, culminating in anemia, prolonged menstrual periods, pelvic compression symptoms, and reproductive challenges. Although their benign nature, they often represent a chronic disease with considerable healthcare costs [9]. The appropriate management of uterine fibroids remains object of debate, and clear consensus guidelines have not yet been established. Patients with asymptomatic or minimally symptomatic fibroids, particularly those of smaller size, may opt for medical management or regular monitoring instead of surgical intervention. However, medical treatments, such as estroprogestins, progestins, or GnRH analogs, while commonly used, are limited by adverse effects over prolonged use [23]. When considering surgical options, the choice is influenced by factors such as the patient’s age, fertility preservation goals, and the preference to avoid extensive procedures. Surgery remains the primary treatment modality for rapidly growing fibroids, suspicion of malignancy, or failed conservative management, with fertility preservation being a priority consideration. The selection of the most appropriate surgical approach remains fundamental in the management of uterine fibroids [24]. Despite advancements in endoscopy and the growing use of the laparoscopic approach, LM continues to represent a minority of all myomectomy procedures performed. LM is considered an intricate procedure necessitating proficiency in multilayer laparoscopic suturing, thereby posing a challenging learning curve. This could be the reason why many surgeons opt for laparotomy when treating symptomatic fibroids, especially in cases of multiple myomectomy. In response to the limitations associated with laparoscopic surgery, robotic surgery has emerged as a potential solution and today represents a procedure that is conventionally performed in many centers around the world. Minimally invasive treatment modalities also mitigate the risk of tissue trauma, post-operative discomfort, and infection, consequently lowering the occurrence of post-operative complications compared to traditional laparotomy surgery [25]. In our center, we use the DaVinci Xi system as a robotic platform. The da Vinci robotic surgical system effectively addresses these challenges posed by difficult laparoscopic procedures and ensures technical proficiency in complex procedures.

Comprising a surgeon’s operative table, a mobile robotic arm, and a 3D imaging system, the da Vinci system offers several advantages. First, its three-dimensional imaging and 10–15 times magnification provide surgeons with a clearer, depth-enhanced view. Second, the system’s EndoWrist laparoscopic instruments offer 7 degrees of freedom, enhancing maneuverability within the abdominal cavity. Third, the robotic system mitigates hand tremors, promoting stability and safety during operations. Moreover, the ergonomic positioning of the operator at the console allows for comfortable execution of complex operations, such as myomectomies, which often entail prolonged operating times, resulting in reduced physical strain [26].

The first robotic myomectomy procedure was performed in 2004 by Advincula et al. [27]. Over the years, advancements in robotic systems, coupled with cost reductions associated with procedures, have facilitated the widespread adoption of RM, which has obtained significant diffusion, particularly in the management of complex cases involving multiple fibroids.

Numerous studies have examined the comparative outcomes of RALM and LM. However, a definitive consensus regarding the advantages of robotic surgery over conventional laparoscopy remains elusive; moreover, the cost of robotics is still higher than that for conventional techniques [28].

A recently published meta-analysis evaluated 15 retrospective studies examining the outcomes and efficacy of RALM versus LM [24]. The results of this meta-analysis showed that the LM group has a shorter operative time compared to RALM group. RALM appeared to be superior in terms of post-operative hospital stay, lower rate of post-operative complications, lower levels of intraoperative bleeding, lower rate of blood transfusion, lower laparotomy conversion rates, and fewer post-operative complications than the LM group. While the data from this meta-analysis may suggest the superiority of robotic technology over conventional laparoscopy in several aspects, it is important to note that all included studies were retrospective. Furthermore, a significant discrepancy arises from the type of center where the comparisons were carried out and from variations in the surgical skills of the operating surgeons.

Considering the relatively recent integration of robotic surgery, it is evident that compared to traditional laparoscopic techniques, there may be a lack of training among robotic surgeons. It is within this framework that our study fits.

Analyzing our primary outcomes, robotic surgery demonstrates better performance in terms of OOT, OTM, and hospital stay compared to LM as shown in Table 2. However, no significant difference was observed concerning pre- and post-operative hemoglobin levels, EBL, and rates of conversion to laparotomy.

These data appear to diverge from the current trend observed in the literature. However, following a meticulous analysis, we attempted to elucidate explanations concerning the notable differences in operative times, particularly evident in the shorter OOT and OTM compared to traditional LM, as illustrated in Table 2. First, many studies included in prior meta-analyses are dated and reflect a period when robotic surgery was not yet widespread and had not attained standardization as it has today. Additionally, the variance in operating times could stem from the varying levels of experience among gynecologists. Introduction of RALM occurred at different times across medical centers, with some delay behind, resulting in a steeper learning curve for surgeons and consequently longer procedure times.

In our institution, the integration of robotic surgery began years ago, initially for oncological procedures and subsequently for benign gynecological surgeries. We have implemented a standardized approach facilitated by a specialized team comprising nurses, surgeons, social health workers, and anesthesiologists proficient in robotic surgery. This comprehensive approach has led to reduced downtime, streamlined processes such as robotic arm dressing and docking, and heightened efficiency among operating surgeons at the console. The focal point therefore remains the standardization of the entire process, always using the same team or at most providing intensive training to young apprentices regardless of the figure involved in robotic surgery.

Experience plays a pivotal role in the duration of RALM procedures. With increased exposure and proficiency, gynecologists can achieve comparable or even faster procedure times compared to laparoscopy. The ease of surgical suturing, particularly evident in cases of multiple myomectomies within a single-, double-, or triple-layer sutures, contributes significantly to the enhanced efficiency of robotic procedures, especially for less-experienced laparoscopic surgeons. In the United States where robotic surgery was widely spread early on, gynecologists who are less experienced in the use of laparoscopy may prioritize or prefer the use of robotic surgical systems, as they find the technology easier to learn [29].

Furthermore, our study did not reveal any differences in pre- and post-operative hemoglobin levels, EBL, or rates of conversion to laparotomy. This may be attributed to the fact that all procedures in our center were performed by the same two highly skilled surgeons (G.B. and P.G) proficient in both laparoscopic and robotic techniques, with extensive experience (> 100 procedures performed). In centers where surgeons favor robotic surgery, complex procedures like multiple myomectomies may exhibit greater surgical precision, reduced bleeding, and consequently, higher post-operative hemoglobin levels, explaining our results compared to LM.

Robotic surgery is often considered “more” minimally invasive due to the smaller incisions in the abdominal wall, typically ranging from 0.5 to 1.2 cm. Compared to laparoscopy, the enhanced precision and dexterity offered by robotic surgery enable finer tissue manipulation, precise movements, and greater maneuverability within confined anatomical spaces. These advantages contribute to mitigating tissue damage during the procedure, thereby reducing intraoperative bleeding, tissue trauma, and ultimately, post-operative hospitalization stays [30]. The less-invasive nature of robotic surgery reduces the likelihood of bleeding, infection, and adhesion formation, thereby lowering the overall complication rate relative to laparoscopic procedures [30]. In terms of post-operative hospital stay, our results, in line with the studies published so far [25], showed a faster recovery in the RALM group. It is noteworthy to underline that shorter hospital stays correspond to reduced costs associated with the surgical procedure. While it is true that robotic surgery procedures typically have greater costs compared to traditional laparoscopic surgery, comprehensive cost-effectiveness analyses should extend beyond, assessing in particular the post-operative hospitalization length. They should include additional factors, such as the positive economic impact associated with an early return to work and daily activities. This aspect requires future investigation and pushes us to reflect on the potential of RALM to achieve cost neutrality or even cost reduction compared to LM [18].

An important aspect regarding the two surgical procedures is that we had no case of endometrial cavity opening during the myomectomy, indicating the clear identification of all intramural myomas at imaging and the great performance of the surgical procedure: this is particularly important in patients who want to become pregnant, because the onset of post-surgical intrauterine adhesions can lead to infertility [31].

The strength of our study rests on the reduction in operating times, hospital stays, and, consequently, potential cost savings; moreover, to limit inter-operator variability, the procedures were performed by only two experienced surgeons. Limitations include the retrospective nature of the study, the small sample size and its heterogeneity in terms of age and BMI. Despite these limitations, our findings contribute new data into the efficacy and safety of RALM compared to LM, stimulating future reflections.

## Conclusion

In conclusion, our study provides valuable insights into the use of robotic-assisted laparoscopy compared to traditional laparoscopy in the surgical management of uterine fibroids. Despite inherent limitations, our findings suggest potential advantages of RALM over LM, particularly in terms of reduced operative times, shorter hospital stays, and potential cost savings. The standardized approach and extensive experience of our surgical team likely contributed to these great results. However, further randomized multi-institutional studies with larger cohorts are warranted to validate our findings and elucidate the broader implications of robotic surgery in gynecological practice. As technology evolves and surgical techniques advance, continuous evaluation and refinement of treatment modalities are essential to optimize patient outcomes and enhance the quality of care in the management of uterine fibroids.

## Data Availability

Data will be made available on request.
